# Safety, Adherence and Acceptability of Intermittent Tenofovir/Emtricitabine as HIV Pre-Exposure Prophylaxis (PrEP) among HIV-Uninfected Ugandan Volunteers Living in HIV-Serodiscordant Relationships: A Randomized, Clinical Trial

**DOI:** 10.1371/journal.pone.0074314

**Published:** 2013-09-26

**Authors:** Freddie M. Kibengo, Eugene Ruzagira, David Katende, Agnes N. Bwanika, Ubaldo Bahemuka, Jessica E. Haberer, David R. Bangsberg, Burc Barin, James F. Rooney, David Mark, Paramesh Chetty, Patricia Fast, Anatoli Kamali, Frances H. Priddy

**Affiliations:** 1 Medical Research Council (MRC)/Uganda Virus Research Institute (UVRI) Uganda Research Unit on AIDS, Entebbe, Uganda; 2 Massachusetts General Hospital and Harvard Medical School, Boston, Massachusetts, United States of America; 3 The EMMES Corporation, Rockville, Maryland, United States of America; 4 Gilead, Foster City, California, United States of America; 5 International AIDS Vaccine Initiative, Nairobi, Kenya; 6 International AIDS Vaccine Initiative, Johannesburg, South Africa; 7 International AIDS Vaccine Initiative, New York, New York, United States of America; Asociacion Civil Impacta Salud y Educacion, Peru

## Abstract

**Background:**

Efficacy of oral pre-exposure prophylaxis (PrEP) in prevention of HIV acquisition has been evaluated using a daily regimen. However, adherence to long term daily medication is rarely perfect. Intermittent regimen may be a feasible alternative. Preclinical studies have demonstrated effectiveness of intermittent PrEP in SHIV prevention among animals. However, little is known about intermittent PrEP regimens.

**Design:**

Seventy two HIV-uninfected volunteers in HIV serodiscordant couple relationships in Uganda were randomly assigned to receive daily oral Tenofovir/Emtricitabine (TDF/FTC-Truvada) or placebo, or intermittent (Monday, Friday and within 2 hours after sex, not to exceed one dose per day) oral TDF/FTC or placebo in a 2:1:2:1 ratio. Volunteers and study staff were blinded to drug assignment, but not to regimen assignment.

**Methods:**

Volunteers were followed for 4 months after randomization, with monthly clinical and laboratory safety assessments and comprehensive HIV risk reduction services. Adherence was monitored using medication event monitoring system (MEMS) and self-report. Sexual activity data were collected via daily short text message (SMS) and self-report. HIV-specific immune responses were assessed by IFN-γ ELISPOT.

**Results:**

Both daily and intermittent oral TDF/FTC regimens were well tolerated. Median MEMS adherence rates were 98% (IQR: 93-100) for daily PrEP regimen, 91% (IQR: 73-97) for fixed intermittent dosing and 45% (IQR: 20-63) for post-coital dosing. SMS response rate was 74%, but increased to 80% after excluding server outages; results may have been affected by the novelty of this measure. The majority of volunteers expressed willingness with no particular preference for either regimen.

**Conclusions:**

Both daily and intermittent oral PrEP dosing regimens were safe. Adherence was high for daily and fixed intermittent dosing; post-coital dosing was associated with poor adherence. Fixed intermittent PrEP regimens may be feasible especially if a minimum effective drug concentration correlating with HIV prevention can be achieved with this dosing.

**Registration:**

Clinicaltrials.gov number NCT00931346

## Introduction

Most adult HIV infections in Africa are due to heterosexual transmission [1], and being in a stable HIV discordant sexual relationship is associated with a 10-fold higher risk of HIV transmission than being in a concordant HIV-negative relationship [2,3]. HIV-uninfected individuals in discordant couple relationships are therefore among the most at risk populations (MARPs). HIV serodiscordant couples enrolled in an HIV vaccine feasibility study in Masaka, Uganda, had an HIV incidence rate of 4.3 and 4.4 per 100 person years (PY) in men and women respectively [4].

At the peak of the HIV epidemic, Uganda adopted the promotion and dissemination of several prevention strategies to control HIV transmission including abstinence, being faithful to one’s partner, reducing the number of sexual partners, treatment of sexually transmitted infections (STI), HIV voluntary counseling and testing (plus sharing of results with partners) and consistent and correct use of condoms [5]. These strategies helped to reduce HIV prevalence [6]; however, they have limitations. HIV prevention programs that focus on reducing the number of sexual partners, use of condoms during casual sex and increased fidelity among married partners are not likely to directly decrease the risk of HIV transmission among persons already living in HIV serodiscordant relationships [3]. Therefore, research into new approaches to HIV prevention particularly in HIV discordant couples remains critical.

Recently, several trials of antiretroviral pre-exposure prophylaxis have shown major reductions in HIV acquisition. In the Pre-exposure Prophylaxis Initiative (iPrEx) study - a randomized multinational clinical trial among men who have sex with men (MSM) - daily fixed dose combination regimen of tenofovir disoproxil fumarate combined with emtricitabine (TDF/FTC), reduced HIV acquisition by 44% overall [7]. Efficacy correlated with adherence and detectable drug levels. Pill use on 90% or more of days was associated with 73% efficacy, while detectable drug levels were associated with 92% efficacy. Subsequent pharmacokinetic modeling of the iPrEx data suggest that 7 days per week dosing could achieve 99% efficacy in prevention of HIV infection among MSM, while 4 days per week could still lower risk by 96% [8]. In the TDF2 study – a randomized trial conducted in Botswana among young HIV-uninfected men and women - daily use of TDF/FTC reduced HIV acquisition by 62% [9]. In the Partners PrEP trial – a randomized multinational trial in HIV discordant couples in Kenya and Uganda - daily PrEP of either TDF alone or combined as TDF/FTC reduced the risk of HIV acquisition by 67-75% [10]. Having detectable plasma tenofovir levels was associated with 86 and 90% reduction in HIV acquisition, for the TDF and TDF/FTC groups respectively [11]. Interestingly, two other randomized PrEP trials in at-risk women failed to find a reduction in risk of HIV infection in the treatment group. The FEM-PrEP trial of oral TDF/FTC in at-risk African women was halted early due a likelihood of being unable to demonstrate difference in HIV seroconversion based on interim data safety monitoring board determination, as was the case for oral and vaginal tenofovir arms of the VOICE study [12,13]. The explanations for these contradictory results are not fully understood; however, low adherence appeared to play an important role. In the FEM-PrEP trial, adherence by self-report and pill counts were high, but plasma drug levels revealed that only 15-26% of samples from HIV seroconverters actually had tenofovir detected, as well as only 26-38% of non-seroconverting controls [14]. Thus, study medication adherence was too low to assess its protective effect in the trial. In 2012, the US Food and Drug Administration approved daily Truvada for HIV prevention in individuals at high risk of sexual HIV infection [15].

The above trials all included daily oral regimens which may be challenging as a population-level HIV prevention method. Daily PrEP may be limited by adherence challenges, as well as acceptability, toxicity and cost concerns, although most of the blinded large efficacy trials have shown it to be acceptable and safe. An intermittent dosing regimen may be an alternative strategy if able to achieve adequate drug levels required for HIV prevention. The biological characteristics of TDF and FTC favour usage of an intermittent PrEP regimen. Both TDF and FTC are phosphorylated to active forms that have long half-lives in plasma and peripheral blood mononuclear cells (PBMCs) of ≥60 and 39 hours respectively [16,17]. Additionally, in studies using macaques as animal models, intermittent PrEP, given 2 hours before and 24 hours after each weekly virus challenge, protected animals against SHIV as well as daily PrEP [18].

In this randomized, double-blind study, we evaluated safety, acceptability and adherence to an intermittent PrEP regimen with TDF/FTC compared to a daily regimen among men and women living in HIV serodiscordant couple relationships. The intermittent regimen was defined as twice weekly dosing (Mondays and Fridays) plus a coitally-dependent dose, but not exceeding one pill per day. This regimen was selected based on primate challenge data described above, which suggested that having steady state drug levels in addition to dosing soon after exposure was important for protection [18].

This study was conducted and completed before the release of any PrEP efficacy studies referenced above. A parallel study with the same design was conducted in Kenyan MSM and female sex workers (FSW) and results published elsewhere [19].

## Methods

The protocol for this trial and supporting CONSORT checklist are available as supporting information; see Checklist S1 and Protocol S1.

### Participants

The study was conducted at the MRC/UVRI Uganda Research Unit on AIDS’ Masaka site in South Western Uganda. The site had an active HIV serodiscordant couple prospective cohort from which volunteers were recruited for this study. In this cohort, every 3 months, volunteers were provided with a comprehensive HIV prevention package including couple HIV/STI testing, and risk reduction counseling, male and female condoms, treatment of STIs, referral for adult medical male circumcision and for ART initiation when indicated per Ugandan national guidelines. Eligible HIV-uninfected volunteers were healthy adults aged 18-49 years in cohabiting HIV serodiscordant relationships who had reported any episodes of unprotected vaginal sex with their partner in the past 3 months and the infected partner not using ART. Volunteers with chronic hepatitis B infection (HBsAg-positive) or with creatinine clearance <80mL/min or pregnant or lactating mothers were excluded from the study due to possible drug toxicity concerns. Women of childbearing potential were required to use a non-barrier form of contraception (hormonal contraception or intrauterine device) during the study. In addition, condoms were provided to all volunteers for protection against HIV and STIs. Both partners in the HIV serodiscordant couple were enrolled in the study, but only the HIV-uninfected partner was randomized to receive study drug. After randomization, volunteers would be discontinued from study medication if creatinine clearance was ≤ 50mL/min by Cockcroft-Gault formula.

### Community stakeholder consultations

The site conducts regular community stakeholder consultations on new and ongoing research and has an active community advisory board (CAB). The CAB reviewed the study aims and patient information materials for the protocol and provided feedback on potential community concerns.

### Ethics

Both members of the couple were aware of each other’s HIV status and gave written informed consent to participate in the study. The study was approved by Uganda Virus Research Institute Science and Ethics Committee (UVRI-SEC), Uganda National Council for Science and Technology (UNCST) and the National Drug Authority (NDA).

### Objectives

The objectives of the study were (1) to evaluate the safety of daily and intermittent dosing of TDF/FTC; (2) to compare the acceptability of and adherence to daily and intermittent regimens; (3) to evaluate changes in HIV-associated risk behaviour; (4) to evaluate HIV-specific immune responses in volunteers randomized to TDF/FTC and placebo.

### Outcomes

The main outcome measures were: (1) clinical adverse events including mild, moderate and greater severity renal toxicities and serious adverse events (2), adherence rates to daily and intermittent dosing (3), willingness to use the study regimen, if shown to be effective (4) change in HIV-associated risk behaviour during trial participation (5), the proportion of volunteers with HIV-specific immune responses as measured by interferon-γ ELISpot.

### Study procedures

Volunteers were randomized to daily TDF/FTC or placebo, or intermittent (fixed dose on Mondays, Fridays and post-coital dose within 2 hours after sex, not to exceed 1 dose per day) TDF/FTC or placebo in a 2:1:2:1 ratio, and were followed monthly with standardized adherence and HIV risk reduction counseling, HIV testing, and safety evaluation for 4 months. HIV risk reduction strategies included provision of male and female condoms, treatment of STIs, referral for adult medical male circumcision and for ART initiation when indicated per Ugandan national guidelines. STI testing was conducted at screening and thereafter whenever infection was suspected. The primary measure of adherence was the medication event monitoring system (MEMS; Aardex, Switzerland), in which a microchip in the cap covering the pill bottle electronically recorded every time the bottle was opened and closed. Data were uploaded monthly. Adherence was also assessed using monthly self-report per a timeline followback calendar [20-22]. In brief, the calendar is used to prompt recall of recent behaviour and has been used successfully in other behavioural studies (e.g. alcohol use). Self-report of openings without removing pills (“curiosity openings”) and of removing multiple pills at one opening (“pocket doses”) were collected each month. Sexual activity data were collected via daily short message service (SMS) text message queries as well as through the timeline followback face-to-face risk assessment with a one-month recall period (collected concurrently with the adherence data). SMS queries prompted volunteers to enter a password and then asked in the volunteers preferred language, ‘Did you have vaginal sex with your main partner in the last 24 hours?’, ‘Did you use a condom?, ‘Did you have vaginal sex with any other partner in the last 24 hours?’, ‘Did you use a condom?’ Volunteers were provided with free mobile phones, SIM cards and call credits for every successful response. Given the novelty of SMS surveys in collecting adherence data, post-coital adherence was also calculated using follow-back self-report data; however, the SMS responses were the primary measure of sexual activity/behaviour and were used to calculate overall intermittent dosing adherence and specifically post-coital adherence. Hematologic and biochemical evaluations were performed monthly. Full details of the trial protocol can be found in the Supplementary Appendix, available with the full text of this article at www.plosone.org.

### Sample size

Seventy-two volunteers were randomized to active daily and intermittent TDF/FTC regimens or respective placebos (24 active and 12 placebo recipients per group). Because this was an exploratory study to evaluate safety, adherence and acceptability of intermittent PrEP, it had limited power to rule-out small differences in safety and adherence. For example, with 24 volunteers assigned to either daily or intermittent TDF/FTC, observing no medication-related serious toxicity would result in an exact, two sided 95% confidence interval of 0-14.3% for the corresponding true incidence. Therefore, observing no medication-related severe toxicities provided 97.5% confidence that the true incidence was no more than 14.3%. With 36 volunteers (combining active and placebo for each regimen), the study had 51% power to detect a true adherence rate of 90% for each regimen. Assuming condoms were used always or frequently (more than half the time) by 60% of volunteers at baseline, the study had >80% power to detect a 50% decrease in condom usage by treatment group (n=24).

### Randomization and blinding

A random allocation sequence was generated by an external data coordinating center. The study product was randomly assigned to volunteers in mixed blocks of 3 and 6, and dosing schedules randomly assigned within study product using a block size of 2. Investigators at the study site enrolled volunteers via an electronic enrollment system, where allocation codes were assigned consecutively to eligible volunteers at the time of first dispensation of study drug. Allocation to TDF/FTC or identical placebo tablet was blinded to study volunteers, all research staff and the study sponsor. Allocation to daily or intermittent dosing was not blinded.

### Laboratory methods

Screening and follow-up clinical laboratory safety procedures were performed on-site following Good Clinical Laboratory Practices (GCLP) in laboratories accredited by Qualogy, UK [23]. Interferon-γ ELISpots were performed on frozen peripheral blood mononuclear cells (PBMCs) at MRC/UVRI laboratories and IAVI Human Immunology Laboratory on baseline and follow-up specimens as described previously [24]. A positive ELISpot was defined as (1) an average background (mock)-subtracted count per peptide greater than 38 spot forming units (SFU) per 10^6^ PBMCs with the coefficient of variation no greater than 70%, (2) mean count greater than 4x mean background (mock), and (3) mean background (mock) below 50 SFU/10^6^ PBMCs.

### Statistical methods and adherence calculations

Comparisons of categorical and continuous variables were conducted using Fisher’s exact test and Wilcoxon rank-sum test, respectively. A two-sided p-value of less than 0.05 was considered to indicate statistical significance. Statistical analyses were performed with the use of SAS software, version 9.2 (SAS Institute, Cary, NC, USA). The following adherence definitions were used:

### Primary analyses

#### Unadjusted monthly MEMS adherence for the daily group

The number of MEMS events in 28 days was divided by 28 days.

#### Adjusted monthly MEMS adherence for the daily group

The number of curiosity openings was subtracted from the number of MEMS openings, while the number of pocket doses was added to the number of MEMS openings. Estimates were calculated over a 28-day interval.

#### Unadjusted monthly MEMS adherence for the intermittent group

The sum of days when volunteers were adherent to fixed dosing (Mondays and Fridays with a MEMS event, and non-Mondays and non-Fridays on which neither sexual activity nor a MEMS event occurred) plus post-coital dosing (other days on which sexual activity was reported by SMS and a MEMS event occurred), divided by 28.

#### Adherence to fixed doses

The number of MEMS events on Mondays and Fridays in a 28-day interval was divided by 8 days.

#### Adherence to post-coital doses

The number of MEMS events on sexual event days in a 28-day interval was divided by the number of sexual event days per SMS.

#### Adherence to post-coital doses within 2 hours of sex

The number of days of post-coital dosing within 2 hours of sex by timeline followback report divided by the number of days with sexual events per SMS.

### Secondary analyses

#### Adjusted monthly MEMS adherence for post-coital dosing in the intermittent group

Intermittent dosing was adjusted for extra pills taken out, with all pills assigned to post-coital dosing.

#### Alternate definitions of post-coital dosing

Because SMS response rates varied (see below), adherence to post-coital doses was also calculated as the number of MEMS events on days with timeline followback reported sexual events divided by the number of days with timeline followback reported sexual events. Adherence to post-coital doses within 2 hours of sex was also calculated as the number of days on which post-coital dosing occurred within 2 hours of sex by timeline followback report divided by the number of days on which sexual events occurred by timeline followback report.

Acceptability of PrEP was assessed by a 4-value Likert scale after 16 weeks in the study. Volunteers with missing responses for acceptability questions were excluded from the denominator.

## Results

### Participant flow

A total of 133 HIV serodiscordant couples were screened, of which 72 (36 HIV-uninfected males and 36 HIV-uninfected females) were enrolled and randomized into the study from October 2009 through March 2010. Sixty-eight volunteers (94%) completed the study (Figure S1). Having abnormal laboratory parameters was the commonest reason for study ineligibility, 34/61 (56%). Among those ineligible for enrollment due to baseline laboratory abnormalities (n=34), creatinine clearance of <80mL/min by Cockcroft-Gault formula and proteinuria on urine dipstick were the most common (77% and 18% respectively). The other major category for exclusion was study being fully enrolled by the time lab results needed for verification of volunteer eligibility became available (n=16). The trial ended when the last enrolled volunteer completed the study follow-up schedule.

### Baseline data

Baseline demographic and HIV risk characteristics were similar among the groups (Table 1). 83% of volunteers had 1 sex partner and the remaining 17% had > 1 sex partner in the past month prior to enrollment. Fifty seven volunteers (79%) reported always using a condom with the HIV-infected partner. Alcohol use before sex and use of street drugs were uncommon.

**Table 1:** Baseline demographics and HIV risk factors for the past 28 days by treatment assignment and treatment schedule.

	**Treatment**					
	**Active**		**Placebo**			
**Variable**	**Daily(24) n(%)**	**Intermittent(24) n(%)**	**Daily(12) n(%)**	**Intermittent(12) n(%)**	**Total(72) N(%)**
**Male gender**	12 (50)	13 (54)	4 (33)	7 (58)	36 (50)
**Age – yr (mean (range))**	33 (20-47)	33 (22-48)	33 (26-47)	33 (27-46)	33 (20-48)
**Drank alcohol before sex**	2 (8)	2 (8)	2 (17)	0	6 (8)
**Used any street drugs**	1 (2)	0 (0)	1 (3)	0 (0)	1 (1)
**Genital sore or discharge**	2 (8)	1 (4)	3 (25)	2 (17)	8 (11)
**Number of sex partners past month**					
**1**	23 (96)	17 (71)	12 (100)	8 (67)	60 (83)
**2**	1 (4)	6 (25)	0	4 (33)	11 (15)
**3**	0	1 (4)	0	0	1 (1)
**Number of HIV-infected partners past month**					
**0**	0	0	0	1 (8)	1 (1)
**1**	24 (100)	23 (96)	12 (100)	11 (92)	70 (97)
**2**	0	1 (4)	0	0	1 (1)
**Condom use with HIV-infected partners**					
**Not Applicable**	0	0	0	1 (8)	1 (1)
**Never**	1 (4)	0	0	0	1 (1)
**Sometimes**	3 (13)	2 (8)	1 (8)	1 (8)	7 (10)
**Frequently**	1 (4)	4 (17)	1 (8)	0	6 (8)
**Always**	19 (79)	18 (75)	10 (83)	10 (83)	57 (79)

### Participants analyzed

Analysis for safety, adherence, acceptability, and change in risk behaviour included all randomized volunteers who were HIV-1 negative at the time of randomization and for whom study medication was dispensed.

### Outcomes and estimation

#### Safety

Ninety-nine percent (227/228) of the non-serious adverse events (AE) were mild or moderate, with 214 (94%) judged unlikely related or not related to study drug. The proportion of volunteers with moderate or above AEs did not differ significantly by regimen (daily: 50%, intermittent: 53%, p=1.00), or treatment groups (active: 44%, placebo: 67%, p=0.08). The proportion with mild or above AEs was also not different by regimen or treatment group (Table 2).

**Table 2:** Number (percentage) of volunteers with AEs categorized by maximum severity experienced, and treatment assignment and schedule.

**Assignment Schedule**		**Maximum AE Severity**				
		**None**	**Mild**	**Moderate**	**Severe**	**Very Severe**
**Active**	**Daily**	2 (8)	12 (50)	10 (42)	0 (0)	0 (0)
	**Intermittent**	1 (4)	12 (50)	11 (46)	0 (0)	0 (0)
	**Overall**	**3 (6)**	**24 (50)**	**21 (44)**	**0 (0**)	**0 (0**)
**Placebo**	**Daily**	0 (0)	4 (33)	8 (67)	0 (0)	0 (0)
	**Intermittent**	1 (8)	2 (17)	8 (67)	0 (0)	1 (8)
	**Overall**	**1 (4)**	**6 (25)**	**16 (67)**	**0 (0**)	**1 (4)**

The percentage of volunteers with gastrointestinal complaints was not significantly higher in the active treatment group (33%) than the placebo group (29%) (p=0.79). Two volunteers on active regimen had elevated serum creatinine, one mild (1.1-1.3 times the upper limit of normal) and one moderate (1.4-1.8 times the upper limit of normal), which resolved spontaneously while continuing study medication. Seven cases of reduced creatinine clearance occurred; five in active and two in placebo recipients, all of which resolved spontaneously without interruption of study medication. No other renal dysfunction was found. One placebo recipient had an isolated neutropenia, graded as very severe, which resolved spontaneously and did not require discontinuation of study medication. There were no drug-related serious adverse events (SAEs) and no HIV infections detected. Three pregnancies occurred resulting in one normal live birth (daily placebo group), one spontaneous abortion at 6 weeks of pregnancy (daily active group), and a molar pregnancy (intermittent placebo group) which was treated and resolved without sequela.

#### Adherence

There was no difference in adherence rates between active and placebo groups, thus these 2 groups were combined for the adherence analyses (Table 3). Median unadjusted MEMS adherence rates were 97% [IQR: 92-100] for daily dosing and 91% [IQR: 73-97] for fixed intermittent dosing (p=0.02), while adherence to any post-coital doses based on sexual events per SMS reporting was much lower than the previous two rates: 45% [IQR: 20-63] (p<.0001). Adherence rates did not differ by gender. MEMS adherence rates did not change when adjusted for curiosity openings when no pills were taken out or when adjusted for extra openings and extra pills taken out. In a post-hoc analysis, intermittent dosing was adjusted for extra pills taken out, as defined above, with all pills assigned to post-coital dosing (which may or may not have been the case). Median MEMS adherence rate for post-coital dosing did not change after this adjustment 46% [IQR: 23-67]. The median number of days per week of PrEP use according to MEMS data was 6.8 [IQR 6.5-7.0] in the daily group and 2.8 [IQR 2.3-3.3] in the intermittent group. Three volunteers required replacement of MEMS caps due to loss or damage during the study; no data was lost.

**Table 3:** PrEP adherence rates for daily and intermittent groups.

**ADHERENCE GROUP**	Adherence parameter	**Active**	**Placebo**	**P-value**	**Overall**
**DAILY ADHERENCE RATE Median % [IQR]**	Overall unadjusted	98 [89-100]	96 [95-99]	0.87	97 [92-100]
	Adjusted1	98 [92-100]	98 [95-99]	0.88	98 [93-100]
**INTERMITTENT ADHERENCE RATE Median % [IQR]**	Overall2	80 [74-86]	78 [67-86]	0.60	80 [73-86]
	Fixed doses	91 [78-102]	88 [69-94]	0.25	91 [73-97]
	Post-coital doses (MEMS events and sexual events per SMS)3	40 [23-58]	53 [15-79]	0.45	45 [20-63]
	Post-coital doses- (MEMS events and timeline follow back self-report sexual events)^4^	39 [29-56]	31 [21-59]	0.58	37 [25-56]
	Post-coital doses within 2hrs (timeline followback self report and sexual events per SMS)5	110 [67-129]	93 [68-113]	0.55	101 [67-129]
	Post-coital doses within 2 hrs (self-report of doses and sexual events per timeline follow-back calendar)6	100 [94-100]	100 [85-100]	0.46	100 [93-100]

^1^ Adjusted monthly MEMS adherence for the daily group was calculated as the number of curiosity openings was subtracted from the number of MEMS openings, while the number of pocket doses was added to the number of MEMS openings, divided by 28.

^2^ Unadjusted monthly MEMS adherence for the intermittent group was calculated as the sum of days when volunteers were adherent to fixed dosing (Mondays and Fridays with a MEMS event, and non-Mondays and non-Fridays on which neither sexual activity nor a MEMS event occurred) plus post-coital dosing (other days on which sexual activity was reported by SMS and a MEMS event occurred), divided by 28

^3^ Adherence to post-coital doses calculated as number of MEMS events on sexual event days in a 28-day interval divided by the number of sexual event days per SMS.

^4^ Adherence to post-coital doses calculated as the number of MEMS events on days with timeline followback reported sexual events divided by the number of days with timeline followback reported sexual events.

^5^ Adherence to post-coital doses within 2 hours of sex calculated as the number of days of post-coital dosing within 2 hours of sex by timeline followback report divided by the number of days with sexual events per SMS.

^6^ Adherence to post-coital doses within 2 hours of sex calculated as the number of days on which post-coital dosing occurred within 2 hours of sex by timeline followback report divided by the number of days on which sexual events occurred by timeline followback report.

#### SMS response rates and sexual activity

The median daily SMS response rate was 74% (range, 0-95), increasing to 80% (range, 0-100) when days with major SMS server outages (>2 hour of network outage per day) were excluded. Major server outages occurred on 8/251 (3.2%) days. Loss of mobile phones was rare with only 2 phones reported as lost during the study. The median number of days per week when sex occurred by SMS reporting was 1.4 (IQR: 1.0-1.9) in the daily group and 1.6 (IQR: 0.8-2.4) in the intermittent group, and by timeline followback interview it was 1.7 (IQR: 1.0-2.3) and 1.7 (IQR: 0.9-2.4) respectively. In secondary analyses in which MEMS adherence was combined with timeline followback data for sexual activity, the median MEMS adherence rate for post-coital dosing was 37% (IQR: 25-56) (Table 3). Using timeline followback adherence with SMS for reporting of sexual activity data, adherence for post-coital doses within 2 hours of sex was 101% [IQR: 67-129]. Using timeline followback data for both adherence and sexual activity data, adherence rate was 100% [IQR: 93-100].

The most common reasons cited for missing a pill dose were: volunteers not having pills with them (6%), being away from home (5%), and forgetting to take pill (5%). There were no differences in reasons for missing a pill dose between daily and intermittent groups. All volunteers denied sharing pills.

#### Acceptability

Ninety-nine percent (71/72) of participants would be willing to use the pill regimen most or all of the time if it was shown to be safe, effective and inexpensive or free. There was no difference in acceptability between daily and intermittent groups (100% vs. 97%), or between active and placebo groups (100% vs. 96%). MEMS acceptability was high with 98% (71/72) reporting it was somewhat or very easy to use.

#### HIV behavior change

The median number of sexual partners in the past month remained at 1 [IQR: 1-1] during the trial. No other HIV risk behaviors reported at baseline changed during the trial (data not shown).

#### HIV-specific immune responses

Minimal HIV-specific immune responses were detected by IFN-γ ELISPOT at baseline; one of 58 (1.7%) volunteers with specimens available for ELISPOT testing had a positive IFN-γ ELISPOT response at baseline, to an Env peptide pool. Three of 50 (6.0%) volunteers with post-baseline ELISPOT data had a response to one or more peptides at a single time point. One volunteer each in the daily and intermittent active treatment groups responded to an Env peptide pool at week 16. One volunteer assigned to the daily placebo treatment group had positive IFN-γ ELISPOT responses to all pools at week 16. The IFN-γ ELISPOT responses were generally low magnitude.

## Discussion

In this study of both daily and intermittent PrEP regimens among HIV serodiscordant couples, intermittent PrEP was found to be acceptable and safe. Adherence to daily and fixed intermittent dosing was high, but significantly lower for post-coital dosing. To our knowledge, this is the first study to report on safety, adherence and acceptability of an intermittent oral PrEP regimen among heterosexual HIV serodiscordant couples.

Both intermittent and daily PrEP regimens were well tolerated, with a similar proportion of volunteers reporting mild and moderate adverse events in each regimen group. This observation is in agreement with reports from other larger oral daily PrEP studies [7,9,10,12,25,26]. There were similar proportions of volunteers with gastrointestinal complaints among active and placebo, and between daily and intermittent groups in the study. This differed from the iPrEx study that reported more frequent moderate (grade 2 and above) nausea and unintentional weight loss in the active group [7]. This difference could have been due to a shorter follow-up period and fewer volunteers involved in the current study. A few volunteers in the active group had self-limited elevated creatinine (mild to moderate), and several volunteers in both active and placebo groups had reduced creatinine clearance. This finding was similar to reports from other PrEP trials [7,9,26].

Median adherence rate as measured by MEMS for daily use was significantly higher than that for fixed doses within the intermittent regimen; whereas adherence to coitally-dependant dosing was considerably worse. Despite the statistical difference between daily and intermittent fixed arm, adherence rate was in the 90’s for each. There was no change in adherence after adjusting for reported curiosity openings or pocket doses. Based on MEMS data, volunteers in the daily group took the pill on almost every day of the week. This high adherence is similar to that reported in Partners PrEP trial (92-99% as measured by clinic-based pill counts, unannounced pill counts and MEMS) [27] – (Haberer in press) and higher than that reported in iPrEx (89-95% by clinic count and self-report) [7]. In contrast, adherence to both the daily and fixed component in the same intermittent regimen was lower among MSM and FSW in a parallel trial in Kenya. Median MEMS adherence rates were 83% (IQR: 63-92) for daily dosing and 55% (IQR: 28-78) for fixed intermittent dosing, while adherence to any post-coital doses was 26% [IQR: 14-50] [19]. The higher adherence in the current study may be due to providing PrEP in the context of stable, socially supported relationships as well as the uninfected partner’s hope of maintaining good health while at the same time preserving their serodiscordant relationship, as proposed within the Partners PrEP study [28]. These factors may be less likely to play a role in the MSM population studied in the Kenyan trial [29].

Intermittent fixed dosing, at least in this discordant couple population, appears to have comparable adherence to daily dosing, suggesting that intermittent PrEP regimens may be feasible in certain populations. However, the number of doses per week necessary for >90% efficacy is likely to be greater than the 2 doses evaluated in this study. Using data from the iPrEx study, pharmacokinetic modeling suggested 97% efficacy for 4 doses per week, which dropped to 76% with 2 doses per week [8]. These data, derived from protection against HIV infection in MSM, may not be readily applicable to heterosexual transmission. Comparable data on efficacy of less than daily dosing for heterosexual transmission are not available yet, but are anticipated from the Partners PrEP study.

Median post-coital adherence as measured by MEMS was low at 45%. Post-coital dosing among MSM and FSW in the Kenyan study was even lower at 26% [19]. These findings are consistent with the incomplete adherence observed with a pre- and post-coital dosing strategy for vaginally administered tenofovir microbicide gel in the CAPRISA 004 microbicide trial. In that study, which observed a 39% reduction in HIV incidence in the tenofovir group compared to the placebo group, the median adherence was 60%, and 42% of the women were classified as having <50% adherence to two doses of gel for self-reported sex acts in the last 30 days [30]. The low post-coital adherence in the current study could have been due to behavior and/or measurement error. It may be easier to remember to take pills daily, because a routine behaviour is established. Unfamiliarity with use of SMS to collect sexual behaviour data coupled with technical difficulties (network outages and a user interface with multiple questions) may have affected post-coital adherence monitoring even though the technology appeared to be relatively well accepted by study volunteers. Quantitative data collected such as reasons for missed pills, travel away from home and pill sharing did not explain why post-coital dosing adherence was lower. Qualitative data from a parallel trial of daily and intermittent PrEP among MSM and FSW in Kenya identified several factors influencing post-coital dosing adherence: alcohol use around the time of sex, moving locations frequently and transactional sex work [28].

Qualitative data collected during focus group and individual interviews in the present trial may be informative and are being prepared for publication.

Measurement of adherence is challenging with self-report often providing overestimates when compared to objective measures, such as electronic monitoring, unannounced home-based pill counts and random drug levels [31-36]. Indeed in this study, adherence to post-coital dosing as measured by the self report calendar and SMS report of sexual events alone (without MEMS) was very high in comparison with MEMS. MEMS adherence, however, is also limited by the fact that it records bottle opening rather than actual medication use. Therefore, the low rate of post-coital pill use as measured by MEMS in this study could reflect low adherence to PrEP or the inaccuracy of the methods used to measure it. Other PrEP trials have also found discrepancies between conventional methods such as self-report and biologic measures [7,34]. Adherence rate in the iPrEx trial as measured by clinic pill count and self report was high at 93%, yet drug levels showed that only 50% of volunteers were actually swallowing the pills. Subsequent analyses found low levels of drug detection, with only 18% of volunteers with drug levels reflective of daily use [37]. In addition, self-report and clinic pill counts were poorly predictive of drug exposure [36].

Testing for drug levels was included in this study and the findings will be published when available. Participants’ actual use of trial interventions is often considerably lower than their reported use hence, raising the issue of social desirability. Low adherence is likely to compromise potential efficacy of the intervention and may make interpretation of trial data difficult; since intent-to-treat efficacy and as-treated efficacy will be different. Low adherence may also limit detection of adverse events therefore, misrepresenting safety concerns.

The main reasons given for missing pills were volunteers not having pills with them while away from home and forgetfulness, and these reasons were similar for both daily and intermittent regimen groups. These reasons are similar to those cited in a phase 2 oral PrEP trial conducted in West Africa [38].

Acceptability of oral PrEP was high with 99% of participants reporting willingness to use the pills most of the time or all the time if PrEP was shown to be safe, effective, inexpensive or free. There was no significant difference between those willing to use intermittent (97%) and daily regimens (100%).

There was no change in HIV sexual risk behaviour during the study. Although it is theoretically possible that individuals may feel protected and abandon condom use and other protective measures, none of the oral or topical PrEP trials to date have observed increased HIV risk behavior that would suggest risk compensation [7,9,10,14,30]. Despite encouraging data from animal studies, we found no evidence of HIV-specific immune responses as measured by a validated IFN-γ ELISPOT in volunteers on PrEP It is possible that a longer duration of PrEP and/or more intensive mucosal exposures are necessary to develop these responses, or that too few volunteers were exposed to HIV in this trial. Alternatively, if PrEP-related protective responses do exist, they may be located in the mucosa and may not be evaluable with T-cell assays in PBMCs such as IFN-γ ELISPOTs.

## Summary

Our study demonstrated that FTC/TDF was safe when taken either daily or intermittently for HIV pre-exposure prophylaxis by HIV-uninfected volunteers living in coupled serodiscordant relationships. Adherence was high to both daily and fixed intermittent dosing, but not for coitally-dependent dosing, the measurement of which may have been imperfect. These data, together with findings from the Kenyan intermittent PrEP trial [19], suggest that post-coital dosing adherence will be difficult to measure accurately. If the low post-coital adherence observed in these two studies is accurate, post-coital dosing is unlikely to be effective in these populations.

The study was limited by the short follow-up period and relatively small number of volunteers which hamper generalizability of results. Volunteers with creatinine clearance <80 ml/min were not eligible for enrollment. Seventy-three percent of ineligible volunteers were excluded for lab abnormalities, with creatinine clearance <80 ml/min accounting for 43% of these exclusions. The percentage of all screened volunteers who were excluded due to decreased creatinine clearance was 20%; this may limit the generalizability of the results in part to populations with high creatinine clearance. Study volunteers were blinded to their active drug or placebo assignment and at the time of conduct of the study, PrEP had not yet been shown to be effective. Adherence, acceptability, risk compensation behavior and other outcomes may differ when PrEP which is known to be effective is available in the community. Volunteers were recruited from a pre-existing HIV serodiscordant couple cohort in which participants received regular couple and individual HIV risk reduction counseling and other preventive measures. Consistent HIV testing and counseling offered during HIV biomedical intervention studies has been perceived by volunteers to affect their risk behaviour in a positive way, by giving them knowledge and awareness of their HIV-negative status, hence added motivation to negotiate and use condoms more regularly [39]. Therefore, their behaviour may not reflect that of the general population.

## Supporting Information

Figure S1 **Flow of participants.**
(TIF)

Checklist S1 **CONSORT Checklist.**
(DOC)

Protocol S1 **Trial Protocol.**
(PDF)
