# Supplementary figures and images for: Safety, Adherence and Acceptability of Intermittent Tenofovir/Emtricitabine as HIV Pre-Exposure Prophylaxis (PrEP) among HIV-Uninfected Ugandan Volunteers Living in HIV-Serodiscordant Relationships: A Randomized, Clinical Trial
Source: PLoS One. 2013 Sep 26;8(9):e74314. doi: 10.1371/journal.pone.0074314 (PMC3784443; doi:10.1371/journal.pone.0074314)

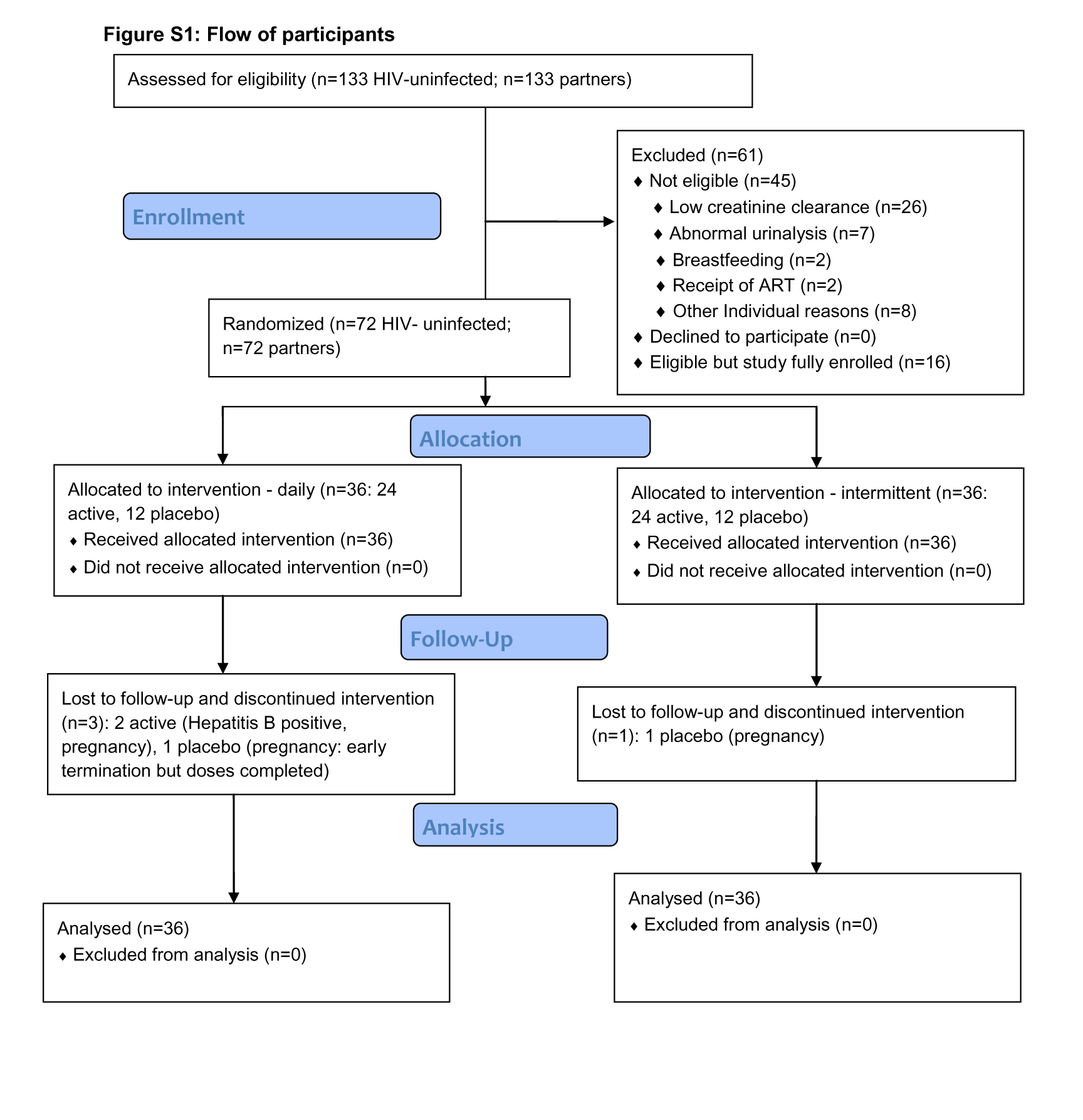

Supplement: Figure S1 — Flow of participants. (TIF) [file pone.0074314.s001.tif]
